# Inhibitory Effect of Progesterone on Breast Cancer Progression and Migration via the Regulation of Epithelial-Mesenchymal Transition

**DOI:** 10.32604/or.2026.071328

**Published:** 2026-03-23

**Authors:** So-Ye Jeon, Zeeshan Ahmad Bhutta, Hong Kyu Lee, Kyung-Chul Choi

**Affiliations:** 1 Laboratory of Biochemistry and Immunology, College of Veterinary Medicine, Chungbuk National University, Cheongju, Chungbuk, Republic of Korea; 2 Department of Companion Animal Health, College of Biomedical Science & Health, Inje University, Gimhae, Republic of Korea

**Keywords:** Progesterone, breast cancer, epithelial-mesenchymal transition

## Abstract

**Objectives:**

Progesterone (P4) is believed to inhibit breast cancer growth, but its role in counteracting estrogen (E2)-driven progression remains unclear. This study aimed to investigate the inhibitory effect of P4 on E2-induced cell proliferation, migration, and invasion in Estrogen receptor (ER)+/progesterone receptor (PR)+ breast cancer cells by examining its regulatory role in the epithelial-mesenchymal transition (EMT).

**Methods:**

ER and PR-positive MCF-7 clonal variant (MCF-7 CV) breast cancer cells were treated with E2 and co-treated with various concentrations of P4. The effects on cell proliferation, migration, and invasion were assessed. The expression of key EMT markers (E-cadherin, N-cadherin, vimentin), transcription factors (Snail, Slug), and apoptosis-related genes (p53, B-cell lymphoma 2 [BCL-2], BCL2-associated X [BAX]) were analyzed.

**Results:**

P4 significantly inhibited E2-induced cell proliferation in a dose-dependent manner. In the presence of E2, P4 treatment reversed EMT characteristics by increasing E-cadherin while decreasing N-cadherin, vimentin, Snail, and Slug. Consequently, P4 inhibited E2-stimulated cell migration and invasion. Furthermore, P4 treatment promoted apoptosis by upregulating BAX and p53 and downregulating BCL-2.

**Conclusion:**

Progesterone can counteract estrogen-driven breast cancer progression in ER+/PR+ cells by inhibiting proliferation, reversing the EMT process, and inducing apoptosis. These findings provide mechanistic insight into the protective role of PR signaling in breast cancer.

## 1 Introduction

In 2022, an estimated 2.3 million new breast cancer cases and 666,000 breast cancer-related deaths occurred globally, accounting for 23.8% and 15.4% of all cancer cases and deaths in women, respectively, making breast cancer the second most common cause of cancer-related mortality worldwide [1]. The malignant tumor originating in breast tissue exhibits uncontrolled growth, with the ability to invade nearby tissue and metastasize to other parts of the body. Early breast cancers may be asymptomatic. However, any of the following changes are known to be conventional symptoms of breast cancer [2]: such as breast lump, breast swelling, changes in breast shape, or nipple discharge [3,4]. Since these signs and symptoms occur when the cancer is in advanced stages, it is essential to detect breast cancer early in women with significant risk factors [5].

Age, heredity, and family history are significant risk factors for breast cancer, with the risk rising as women age. Women aged 55 and above contribute to approximately half of all instances of invasive breast cancer. A greater incidence of breast cancer has been observed in women whose close relatives or immediate family members have already been diagnosed with the illness. Additionally, it is believed that faulty genes passed down from parents cause 5%–10% of breast cancers in offspring [6]. Obesity, lack of physical exercise, alcohol consumption, cigarette smoking, and exposure to chemicals in the environment are some of the known lifestyle-related risk factors for breast cancer [7].

Estrogen, a primary female sex hormone, is a crucial risk factor for breast cancer as it performs a role in controlling the growth and specialization of breast epithelial cells by engaging with two nuclear Estrogen receptors, ERα and ERβ [8,9]. Early menarche, late menopause, and first birth at an older age or never having given birth are considered to be linked with increased breast cancer risk, and this suggests a close correlation between estrogen exposure and breast cancer development [10,11]. Hormone replacement treatment (HRT) raises the risk of breast cancer by approximately 60%–85% [12], even with short-term usage, and the risk is substantially higher with a combination of high doses of HRT compared to moderate doses. Although the mechanisms by which estrogen promotes breast cancer are complex and still incompletely characterized, it plays a crucial role in breast cancer through the estrogen receptors (ERs) that act as transcriptional factors to regulate several target genes directing cell proliferation, growth, motility, and the invasion of cancer cells [13,14]. In addition, other studies have indicated that estradiol accelerates the growth and metastatic potential of breast cancer via non-ER-mediated mechanisms [15,16].

On the other hand, progesterone, another sex steroid hormone in the female reproductive system, is believed to affect cancer growth opposite to that of estrogen. However, this is still controversial and remains to be entirely determined [17]. Progesterone has been shown to inhibit the proliferation of normal breast epithelial cells and T-47D breast cancer cells by inducing apoptosis [18]. Additionally, it has been demonstrated to decrease the proliferation of T-47D breast cancer cells by increasing transcription of the p27 gene, an inhibitor of the cyclin-dependent kinase (CDK) pathway, thus inducing G1 cell-cycle arrest [19]. Also, a previous survey concluded that the progesterone levels at the time of breast cancer surgery correlated with the better survival of node-positive patients [20]. Like ERs, Progesterone receptors (PRs) are ligand-activated nuclear transcription factors. They mediate the biological effects of progesterone. They exist as two isoforms, PR-A and PR-B, which have different physiological functions, with PR-A generally acting as a transcriptional repressor and PR-B functioning primarily as a transcriptional activator. These differences in their roles make it important to distinguish between the two isoforms in breast cancers [17].

This study aimed to investigate the protective role of progesterone against estrogen-induced breast cancer by targeting epithelial-mesenchymal transition (EMT), a critical process in metastasis. Using ER/PR-positive MCF-7 CV cells, we examined the opposing effects of estrogen and progesterone on cell migration, invasion, and the expression of key EMT markers (E-cadherin, N-cadherin, and vimentin).

## 2 Materials and Methods

### 2.1 Reagents and Chemicals

E2 (17β-estradiol) (Sigma-Aldrich, D44679, St. Louis, MS, USA), P4 (Progesterone) (Sigma-Aldrich, P8783), and RU486 (Mifepristone) (Sigma-Aldrich, M8046) were diluted in the dimethyl sulfoxide (DMSO) (Junsei, 35535-3630, Tokyo, Japan). Based on preliminary dose-response experiments, a concentration of 10^−8^ M (10 nM) for RU486 was selected as the optimal dose to effectively inhibit the progesterone receptor without independently affecting cell viability, and this concentration was used consistently in all relevant experiments. A concentration of 0.1% DMSO was maintained for the final treatment of the cells.

### 2.2 Cell Culture and Media

The human MCF-7 CV breast cancer cell line was provided by Dr. K. S. Korach (National Institute of Environmental Health Sciences, NIH, Research Triangle Park, NC, USA). This cell line was authenticated by Short Tandem Repeat (STR) profiling, as described in the original characterization publication. The cells were routinely tested for mycoplasma contamination using a PCR-based assay [21] and were confirmed to be negative throughout the course of the study. This cell line was identified to express both ER and PR at the protein level [22]. Dulbecco’s Modified Eagle’s medium (DMEM) (HyClone Laboratories, SH30022.01, Logan, UT, USA), supplemented with 10% heat-inactivated fetal bovine serum (FBS; Gibco, A5256701, Waltham, MA, USA), HEPES (1%) (Gibco, 15630080), 200 mg/mL streptomycin, and 200 U/mL penicillin G (Gibco, 15140122), was used for the cell maintenance at 37°C in a humidified environment with 5% CO_2_. Furthermore, to mitigate the estrogenic effects, a combination of 5% charcoal-dextran treated FBS (CD-FBS) (HyClone Laboratories, SH30068.02), and phenol red-free DMEM (HyClone Laboratories, SH30585.02) was utilized. A solution of 0.05% trypsin-0.02% ethylenediaminetetraacetic acid (EDTA) was used in cell passages. The media was replaced every 2 days.

### 2.3 Cell Viability Assay

Chemical compounds undergoing experimental evaluation were tested to assess their ability to promote the proliferation of MCF-7 CV cells through the application of the 3-(4,5-dimethylthiazol-2-yl)-2,5-diphenyltetrazolium bromide (MTT; Sigma-Aldrich, 88417) methodology, which is a commonly utilized approach in cell viability assays. A total of 4 × 10^3^ cells were cultured in each well of 96-well plates. After the attachment of the cells to the cell surface, the cultured media was replaced with the media containing the test compounds, i.e., E2 and P4. Cultured plates were incubated for 6 days, and the media was changed daily until day 6. Following 6 days, the growth media was discarded entirely. Subsequently, MTT solution (final MTT working concentration of 0.5 mg/mL) was introduced and incubated for 4 h at 37°C in the presence of CO_2_. Finally, the media was removed after incubation and replenished with 100 μL of DMSO in each well before detecting dissolved formazan in an enzyme-linked immunosorbent assay (ELISA) reader (Epoch; BioTek, Winooski, VT, USA) at 540 nm. The cell viability was calculated as per the following formula.

$$Cell\;Viability\;Percentage(\%)=\frac{OD_{Treated\;Sample}-OD_{Blank}}{OD_{Control\;Sample}-OD_{Blank}}\times 100$$

### 2.4 Total RNA Extraction

Over a period of 48 h, the MCF-7 CV cells underwent exposure to E2 (10^−8^ M) in a control group, as well as in conjunction with either RU486 (10^−8^ M) or P4 (10^−8^ and 10^−6^ M). To extract the Total RNA, TRIZOL reagent (Invitrogen Life Technologies, 15596026, Waltham, MA, USA) was added to the cells at 1 mL per 10 cm^2^ of culture dish at the 24-h time point. An ELISA reader (BioTek, USA) was used in conjunction with a Take 3TM Micro-volume plate to determine the concentration of RNA at a wavelength of 260/280 nm. To facilitate the synthesis of cDNA, 1 μg of total RNA was added to diethylpyrocarbonate deionized water (DEPC-DW).

### 2.5 cDNA Synthesis and Reverse Transcription (RT)-Polymerase Chain Reaction (PCR)

The development of cDNA from 1 μg total RNAs was carried out in a final reaction volume of 20 μL by mixing the following agents: 200 pM nonamer random primer (Takara, Shiga, Japan), RNase inhibitor (iNtRON Biotechnology, 25011, Seongnam, Republic of Korea), RT buffer (iNtRON Biotechnology, 25011), dNTPs (iNtRON Biotechnology, 25011), murine leukemia virus reverse transcriptase (M-MLV RT; Invitrogen, 28025021). After 1 h of cDNA synthesis at 37°C, the enzymatic process was stopped by heating the mixture to 95°C for 5 min. RT-PCR was used to analyze the mRNA levels of genes associated with cell cycle and EMT of the MCF-7 CV cells. The reaction product was a mixture of 10× PCR buffer, Taq polymerase, cDNA template, dNTP, and forward and reverse primers (Table 1). EtBr-prepared PCR products were loaded onto an agarose gel (1.5%), and the bands were compared using 100-bp ladders. Gel Doc 2000 (Bio-Rad Laboratories, 170-8110, Hercules, CA, USA) software was used to analyze the bands. The endogenous control for normalization was GAPDH.

**Table 1:** Oligonucleotide sequences of the semi-quantitative reverse transcription-polymerase chain reaction (RT-PCR) products.

Target Genes	Primer Sequence (5^′^-3^′^)	
**E-cadherin**	Forward	CGGGAATGCAGTTGAGGATC
	Reverse	AGGATGGTGTAAGCGATGGG
**Vimentin**	Forward	GAGAACTTTGCCGTTGAAGC
	Reverse	GCTTCCTGTAGGTGGCAATC
**Snail**	Forward	TAACGCCTGACTCTGCTTTT
	Reverse	TTTCGAGCCTGGAGATCCTT
**Slug**	Forward	CCTTCCTGGTCAAGAAGCAT
	Reverse	CACAGTGATGGGGCTGTATG
**BAX**	Forward	TTTGCTTCAGGGTTTCATCC
	Reverse	CAGTTGAAGTTGCCGTCAGA
**BCL-2**	Forward	ACAACATCGCCCTGTGGATG
	Reverse	ATAGCTGATTCGACGTTTTG
**PR**	Forward	CCATGTGGCAAATCCCACAGGAGT
	Reverse	CGGAAATTCCACAGCCAGTGCC
**GAPDH**	Forward	ATGTTCGTCATGGGTGTGAACCA
	Reverse	TGGCAGGTTTTTCTAGACGGCAG

### 2.6 Protein Extraction and Western Blot Analysis

MCF-7 CV cells treated with E2, P4, and RU486 for 48 h were used for protein quantification using western blot. For the extraction of proteins from the chemically treated cells, a RIPA lysis solution maintained at pH 8.0 was used containing the following ingredients: 0.5% deoxycholic acid, 150 mM NaCl (Sigma Aldrich, S9888), 0.1% SDS (Sigma Aldrich, L3771), 50 mM Tris-HCl (Sigma Aldrich, 10812846001), and 1% NP-40 (Sigma-Aldrich, NP40). To quantify the proteins for the western blot analysis, bicinchoninic acid (BCA) assay (Thermo Scientific, 23221, Waltham, MA, USA) was used, and 50 μg of protein was loaded per lane. Prior to antibody incubation, membranes were blocked with 5% bovine serum albumin (BSA, Sigma-Aldrich, A9647) for 1 h. As stated in Table 2, the PVDF membranes were incubated with different primary antibodies at 4°C overnight. Furthermore, the detection of primary antibodies was accomplished by employing the HRP-conjugated anti-mouse IgG (1:2000, Thermo Scientific, 31430) or anti-rabbit IgG (1:2000, Thermo Scientific, A16098) for 2 h at room temperature. Each trial was conducted three times, and the protein bands were quantified using the Gel Doc 2000 machine (Bio-Rad) after treating PVDF membranes with West-Q Chemiluminescent Substrate Plus kit (GenDEPOT, W3651-050, Katy, TX, USA). The levels of all the proteins stated above were adjusted using the GAPDH protein.

**Table 2:** List of primary antibodies used in the study.

Antibody	Host	Catalog Number	Dilution	Company
monoclonal anti-GAPDH	mouse	ab9482	1:1000	Abcam, Cambridge, UK
monoclonal anti-N-cadherin	mouse	ab98952	1:1000	Abcam
polyclonal anti-E-cadherin	rabbit	ab308347	1:1000	Abcam
monoclonal anti-*Snail*	mouse	3895S	1:1000	Cell Signaling Technology, Danvers, MA, USA
monoclonal anti-*Slug* antibody	mouse	ab51772	5 μg/mL	Abcam
polyclonal anti-cathepsin B	rabbit	sc-365558	1:1000	Santa Cruz Biotechnology, Dallas, TX, United States
monoclonal anti-cyclin E1	mouse	ab238081	2 μg/mL	Abcam
monoclonal anti-cyclin D1	rabbit	ab190564	1:1000	Abcam
monoclonal anti-p21	mouse	2946S	1:1000	Cell Signaling Technology
monoclonal anti-p27 antibody	rabbit	ab32034	1 μg/mL	Abcam
monoclonal anti-MMP-9	rabbit	ab137867	1:1000	Abcam

### 2.7 Scratch Assay

MCF-7 CV cells were seeded in 6 well plates at the density of 8 × 10^5^ cells/well and cultured for two days to cover the surface of the plate completely (80%~90%). After that, a scratch was created vertically across the diameter of the well (with the help of 200 μL pipette tips), and the well was rinsed twice with PBS to remove the debris. Later, the cells were treated with DMSO, E2 (10^−8^ M), P4 (10^−8^ and 10^−6^ M), and RU486 (10^−8^ M) in 5% CD-FBS phenol-free media. This time was taken as 0 h, and the micrograph was captured. Similarly, after 48 h, the same regions were photographed again to calculate the proportion of the scratched region that had not yet healed. The uncovered regions were then quantified using the eXcope Lite software (version 3.7.12277; Dixi Science, Daejeon, Republic of Korea). Furthermore, following formula was used to quantify the data obtained.

$$Uncovered\;area\left(\%\right)=\left(\frac{Area_{48h}}{Area_{0h}}\right)\times 100$$

### 2.8 Invasion Assay

A total of 1 × 10^5^ cells were seeded in the upper chamber of the transwell in 24 well plates for two days with a 5% CD-FBS phenol-free medium. In these plates, the transwell upper chamber was coated with Fibronectin (250 μg/mL; Sigma-Aldrich, 11080938001) to trap invading cells from moving to the lower chamber. After that, the cells were replenished with the media containing chemicals under trial (treated with DMSO, E2, P4, and RU486). After rinsing each well with PBS, cells that hadn’t moved into the transwell membrane were scraped from the top side. Finally, the cells were stained with 200 ng/mL of DAPI (Invitrogen, D3571, Carlsbad, CA, USA) for 10 min at 37°C. A fluorescent microscope (Nikon TI-U, Nikon, Japan) was used to analyze the DAPI-stained cells.

### 2.9 Immunocytochemistry

Gelatin pre-coated 6 well plates (0.1% (w/v) gelatin for 30 min at 37°C) were used to seed the MCF-7 CV cells at a density of 5 × 10^5^ cells/well for immunocytochemistry analysis. After the cells were attached completely, the medium containing DMSO, E2, P4, and RU486 was changed for treatment for 48 h. Later, the cells were immobilized using 3% formaldehyde for 10 min, after which they permeabilized using 0.1% Triton X-100 for 10 min at room temperature. Subsequently, the cells were washed thrice with PBS, after which a solution comprising 3% BSA (Sigma-Aldrich, A9647) in PBS-T was administered for 1 h to prevent any potential non-specific reactions. The cells were incubated with the rabbit monoclonal antibody to PR (1:100; Abcam, ab16661) at room temperature for 2 h. Finally, the detection of primary antibodies was accomplished by employing fluorescently labeled secondary antibodies (Alexa Fluor® 488-conjugated anti-rabbit IgG; Abcam, ab150077 or Alexa Fluor® 488-conjugated anti-mouse IgG; Abcam, ab150113) at a dilution of 1:1000 for 1 h at room temperature. Nuclei were counterstained with DAPI (Invitrogen, D3571). The color detection was achieved via laser excitation of these fluorophores (Green channel) and DAPI counterstaining (Blue channel). The images were captured by confocal microscopy (Nikon TI-U, Nikon).

### 2.10 Data Analysis

The data presented in this study are expressed as the mean ± SD, with the experiments being conducted a minimum of three times to ensure reliability and reproducibility. A one-way analysis of variance (ANOVA) followed by Dunnett’s multiple comparison test was employed to analyze the statistical significance of the results. GraphPad Prism (version 7.0) (GraphPad Prism Inc., San Diego, CA, USA) was used to analyze the data. The numerical values reported in the study represent the means ± SD from at least three independent experiments, which are then compared to the control group for any significant differences. The significance levels are denoted by *p*-values of less than 0.05 (*), less than 0.01 (**), and less than 0.001 (***).

## 3 Results

### 3.1 Anti-Estrogen Effects of P4 on the Proliferation of MCF-7 CV Cancer Cells

**Figure 1:**
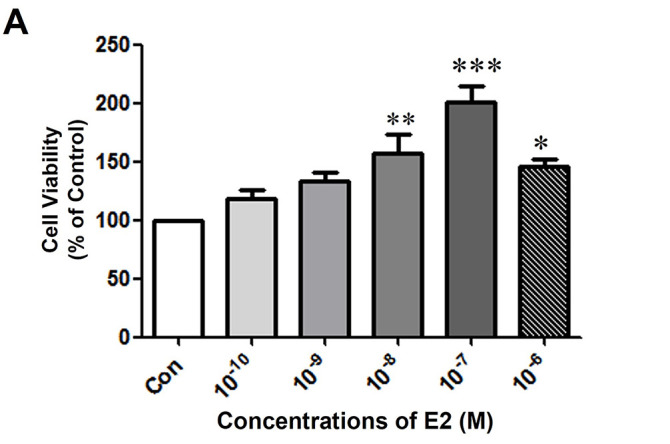

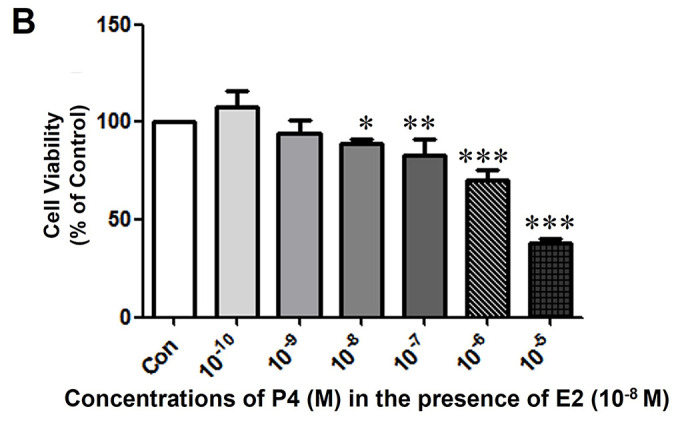
The effects of E2 and P4 on cell proliferation of MCF-7 CV cells. MCF-7 CV cells were seeded at 5000 cells/well in 96-well plates in the presence of phenol red-free Dulbecco’s Modified Eagle’s medium (DMEM) (+5% charcoal-dextran treated FBS [CD-FBS]). After 2 days, the medium was replaced by phenol red-free DMEM (+5% CD-FBS) containing control (0.1% DMSO) and (**A**) E2 at the indicated concentrations for 4 days. (**B**) P4 at the indicated concentrations was co-treated with E2 (control, 10^−8^ M) for 4 days. The cell viability was determined by MTT assay. Values are presented as the mean ± standard deviation (SD) from at least three independent experiments. Asterisks denote a statistically significant difference compared to the control group (*p < 0.05, **p < 0.01, and ***p < 0.001).

To investigate the hormonal interplay in breast cancer, we utilized the MCF-7 CV cell line, which is an established model confirmed to express both ER and PR. The ability of E2 and P4 to affect the proliferation of MCF-7 CV cells was evaluated first to determine the appropriate concentration of E2 and P4 for further experimental analysis. MTT assay was used to evaluate the effectiveness of E2 on breast cancer cell proliferation after being treated for six days. The number of cells increased significantly after treating the cells with E2 compared to the control group (Fig. 1A). The highest activity of E2 was recorded at a concentration of 10^−7^ M. Next; we evaluated whether the increased cell proliferation due to E2 is altered with the P4 co-treatment. Hence, the cancer cells were treated and co-treated with different concentrations of P4 and E2 at a concentration of 10^−8^ M. P4 was recorded to inhibit the effect of E2 on cell proliferation in a dose-dependent manner up to a concentration of 10^−5^ M (Fig. 1B).

### 3.2 Effect of E2 on EMT Induction

**Figure 2:**
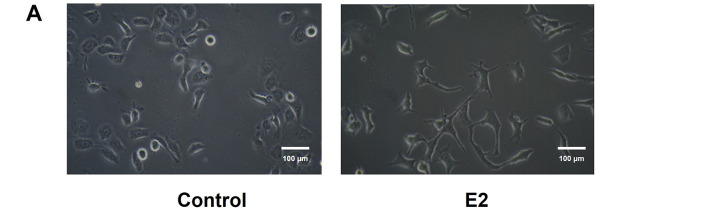

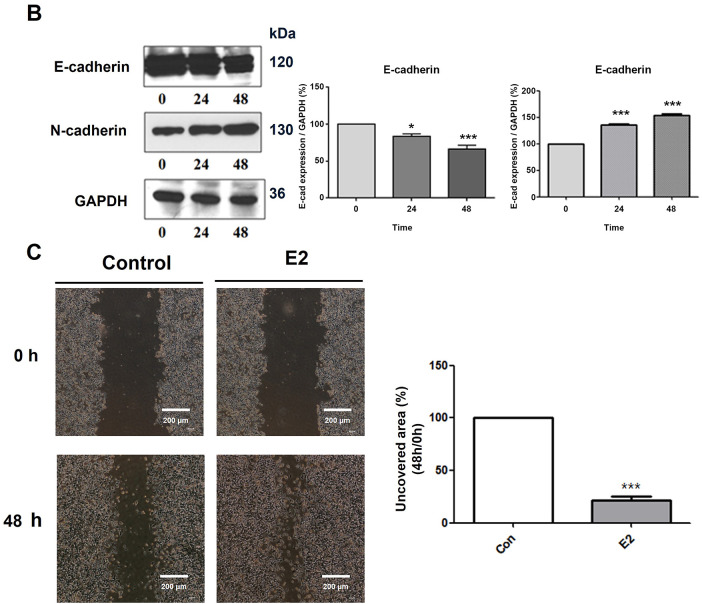
Functional role of E2 in epithelial-mesenchymal transition (EMT) on MCF-7 CV breast cancer cells. MCF-7 CV cells were treated with 0.1% DMSO as a vehicle or (**A**) E2 (10^−8^ M) for 48 h. Morphological changes were observed by microscope under ×20 magnifications. (**B**) The effects of E2 (10^−8^ M) on the expression of epithelial and mesenchymal cell markers in MCF-7 CV cells by western blot were investigated. GAPDH was used as a loading control. Data were quantified using Gel Doc 2000. (**C**) Altered cell migration by the treatment of E2 was investigated using scratch assay. Each image was observed by microscope under ×4 magnifications. Quantification of uncovered areas was conducted by using an eXcope Lite program. All experiments were done at least three times. Values are presented as the mean ± SD from at least three independent experiments. Asterisks denote a statistically significant difference compared to the control group (*p < 0.05 and ***p < 0.001).

Cancer cells treated with E2 (10^−8^ M) for 48 h revealed apparent morphological changes compared to the control group (Fig. 2A). Phenotypic changes were observed from cuboidal epithelial morphology to spindle-shaped mesenchymal cell-like morphology. Furthermore, these morphological changes were accompanied by changes in the expression of EMT markers and resulted in the decrease of E-cadherin while increasing the N-cadherin in a time-dependent manner (Fig. 2B). This result indicates the role of E2 in the EMT process of these ER-positive breast cancer cells. Furthermore, the MCF-7 CV cells’ migratory ability was increased significantly compared to the control group after 48 h in the scratch wound healing assay, as the scratched area healed faster in the E2-treated group than in the control group (Fig. 2C). Consequently, these results suggest that E2 enhances the EMT potential of ER-positive breast cancer cells along with the migratory activity.

### 3.3 P4 Treatment Altered the Expression of EMT-Related Gene and Protein Levels

**Figure 3:**
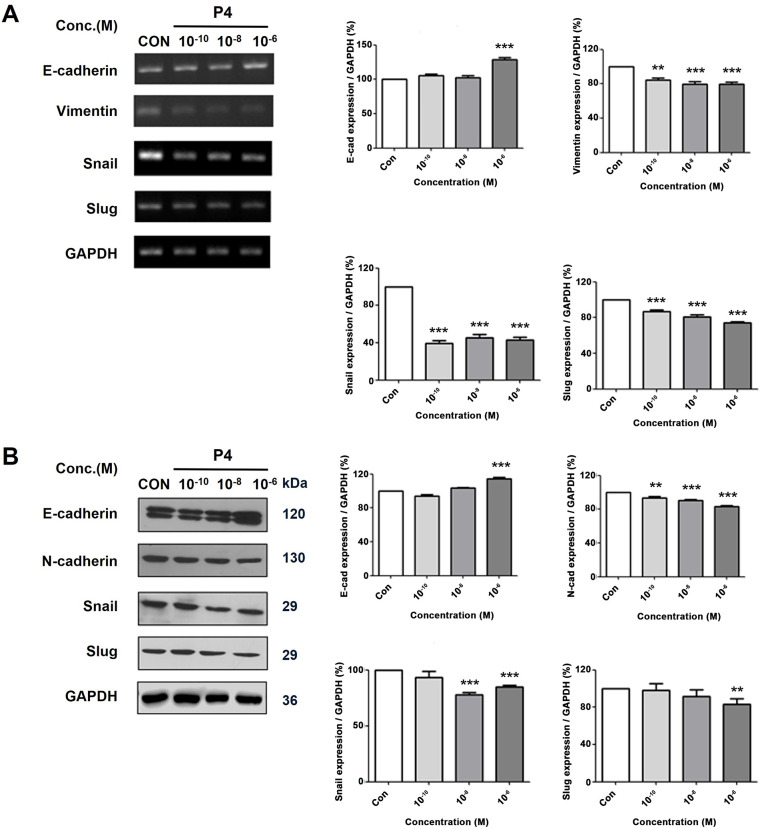
Altered mRNA and protein expressions of EMT-related genes following various concentrations treatment of P4 with E2. In all experiments, MCF-7 CV cells were treated with E2 (10^−8^ M) as a control in combination with P4 (10^−10^, 10^−8^, and 10^−6^ M). (**A**) The level of mRNA expression was investigated through RT–PCR after 24 h of treatment, and (**B**) the protein expression level was investigated by western blot after 48 h of treatment. Data show the alterations in EMT-specific markers (E-cadherin, Vimentin, and N-cadherin) and EMT regulator genes (*Snail* and *Slug*) in MCF-7 CV cells by reverse transcription-polymerase chain reaction (RT-PCR) and western blot. All experiments were done at least three times. Data were quantified using Gel Doc 2000. Values are presented as the mean ± SD from at least three independent experiments. Asterisks denote a statistically significant difference compared to the control group (**p < 0.01 and ***p < 0.001).

To elucidate the role of P4 in the EMT, MCF-7 CV cells were subjected to co-treatment with P4 (at various concentrations) and E2 (10^−8^ M). P4 co-treatment inhibited the E2-induced proliferation of cancer cells. Furthermore, the expression E-cadherin was increased compared to N-cadherin and vimentin, which decreased after P4 treatment. The results were the same for both the mRNA and protein analysis (Fig. 3A,B). Furthermore, the investigation also explored the modification of transcription factors that regulate EMT and cell-specific indicators. Similarly, the expression of Snail (E-cadherin regulator) and Slug (EMT regulator) were decreased after treatment of cells with P4. The data suggest that in this estrogenic environment, P4 modulates the expression of the transcriptional factors Snail and Slug, which are known to regulate markers such as E-cadherin, vimentin, and N-cadherin.

### 3.4 P4 Treatment Altered Cell Migration Ability

**Figure 4:**
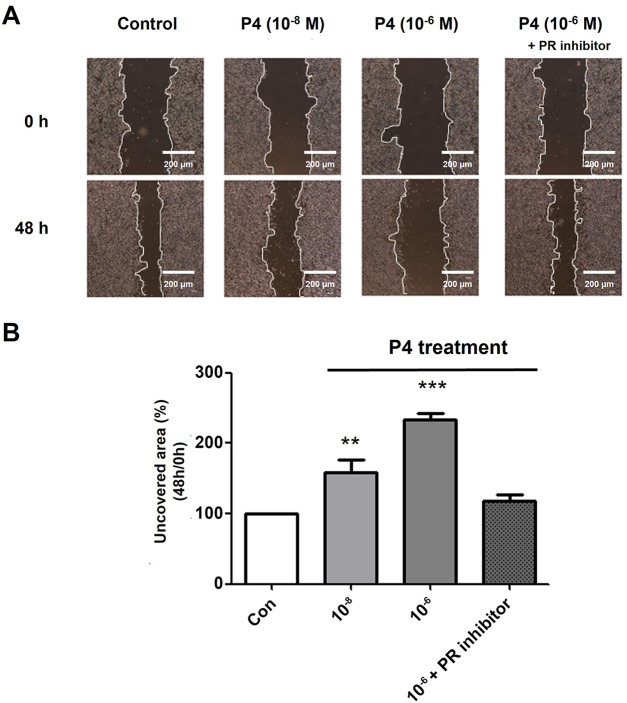
Altered cell migration activity by treating P4 with E2 and the roles of PR. MCF-7 CV cells were treated with E2 (10^−8^ M) as a vehicle (control). (**A**) Altered cell migration by the co-treatment of P4 and RU486 was investigated using scratch assay. Each image was observed by microscope under 4× magnifications. (**B**) Quantification of uncovered areas was conducted by using an eXcope Lite program. Values are presented as the mean ± SD from at least three independent experiments. Asterisks denote a statistically significant difference compared to the control group (**p < 0.01 and ***p < 0.001).

Cancer cells in which EMT is induced are known to have increased migration activity [23]. The effects of the co-treatment of P4 at increasing doses and E2 (10^−8^ M) on cell migration were observed through the scratch assay. To confirm the movement of cells, the area uncovered was recorded after treating the wells for 48 h. The migration activity was reduced after the treatment with P4 at two concentrations (10^−8^ and 10^−6^ M) as seen in (Fig. 4A,B). The results show that P4 inhibits the migratory propensity of cancer cells, and in particular, MCF-7 CV cells did not show migration activity when treated with high concentrations of P4 (10^−6^ M) for 48 h compared with the control group. In addition, the progesterone receptor inhibitor (RU486) was used to inhibit the effects of P4. The results confirmed that the migration activity was restored to the control level. These results confirm that P4 inhibits the migratory ability induced by E2, and that this inhibitory action of P4 is dependent on the progesterone receptor.

### 3.5 P4 Treatment Altered Cell Invasion Ability

**Figure 5:**
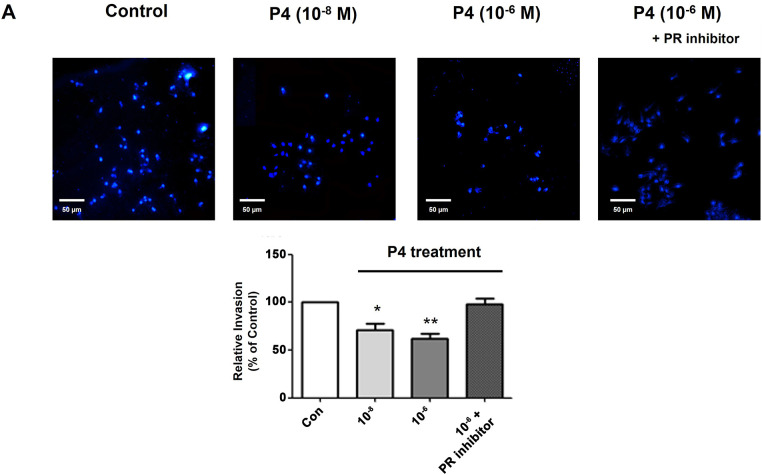

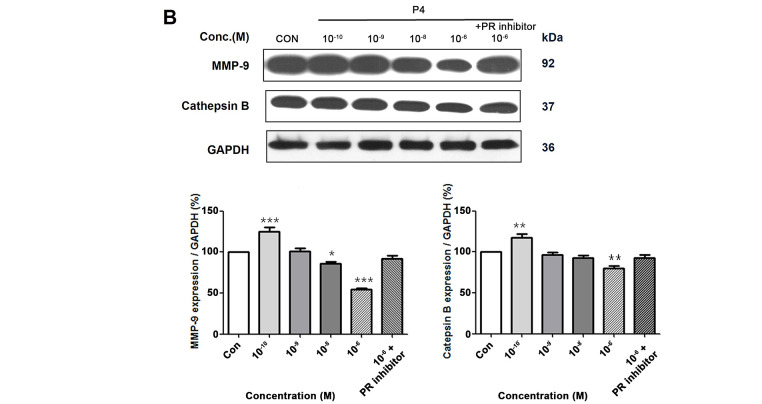
Altered cell invasion activity and protein expressions of migration-related genes by treating P4 with E2. MCF-7 CV cells were treated with E2 (10^−8^ M) as a vehicle (control). (**A**) Altered cell invasive propensity by the co-treatment of P4 and RU486 was investigated using a pre-coated transwell assay. Each image was observed by microscope under 10× magnifications. Data was quantified using ImageJ software. (**B**) The effects of P4 were investigated on the expression of migration-related genes in MCF-7 CV cells by western blot. GAPDH was used as a loading control. Data was quantified using Gel Doc 2000. Values are presented as the mean ± SD from at least three independent experiments. Asterisks denote a statistically significant difference compared to the control group (*p < 0.05, **p < 0.01, and ***p < 0.001).

Cancer cells are known to have the propensity to be more aggressive when the EMT process is induced. Recently, the idea of EMT has been extended to include its role in cancer progression and metastasis. This involves the escape of individual tumor cells from the primary tumor, invasion into nearby tissues, entry into blood vessels, survival in the bloodstream, attachment to and exit from the vessel, and forming a new tumor at a secondary location [24]. An invasion assay was performed to investigate the effects of P4 on cell invasion ability, and a fluorescent microscope was employed to count the number of migrated cells in the lower chamber of the Transwell (Fig. 5A). Similar to the scratch assay results, the P4 treatment (10^−6^ M) decreased migrated cells. In addition to these functional changes, P4 affected the expression of the metastasis-related protein markers. MMP-9 and cathepsin B have a crucial role in cancer migration through the degradation of the extracellular matrix [25]. P4 treatment decreased MMP-9 and cathepsin B expression, which was restored to the normal level when co-treated with the PR inhibitor (RU486) (Fig. 5B). These results suggest that P4 could inhibit cancer migration by modulating the expression of EMT and associated protein markers.

### 3.6 Effects of P4 on the Expressions of Cell Cycle-Related Genes

**Figure 6:**
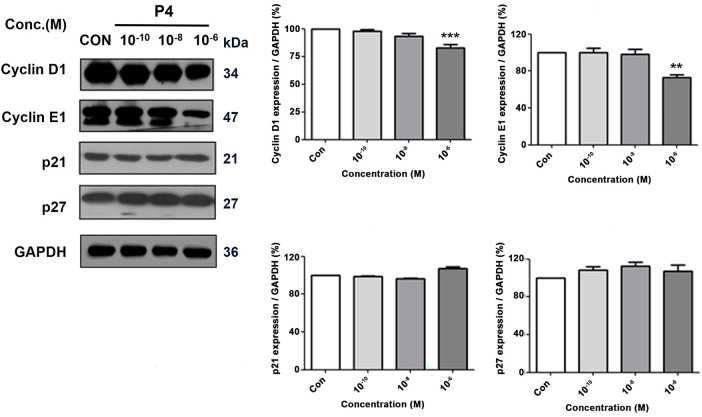
Altered protein expressions of cell cycle-related genes following the treatment of P4 with E2. In all experiments, MCF-7 CV cells were treated with E2 (10^−8^ M) as a control in combination with P4 (10^−10^, 10^−8^, and 10^−6^ M). The protein expression level was investigated by western blot after 48 h treatment. Data show the alterations in protein expression of cell cycle progression genes, cyclin D1 and cyclin E1, and regulator genes of cell cycle progression, p21 and p27, in MCF-7 CV cells. Data was quantified using Gel Doc 2000. Values are presented as the mean ± SD from at least three independent experiments. Asterisks denote a statistically significant difference compared to the control group (**p < 0.01, ***p < 0.001).

To assess and analyze the intricate mechanisms responsible for mediating the impacts of P4 on the cellular proliferation process of the MCF-7 CV cancer cells, a comprehensive examination was conducted to ascertain the changes in the translational expressions of the genes associated with the regulation of the cell cycle. Results revealed that the P4 (10^−6^ M) treatment increased the regulator genes of cell cycle progression (p21 and p27) while decreasing the cell cycle progression (cyclin E1 and cyclin D1) (Fig. 6). While P4 treatment appeared to increase p21 and p27 levels, these changes were not statistically significant, which leads to the fact that P4 does not directly exert a cytotoxic effect on cancer cells but partially regulates cell proliferation.

### 3.7 Effects of P4 on the Expressions of Apoptosis-Related Genes

**Figure 7:**
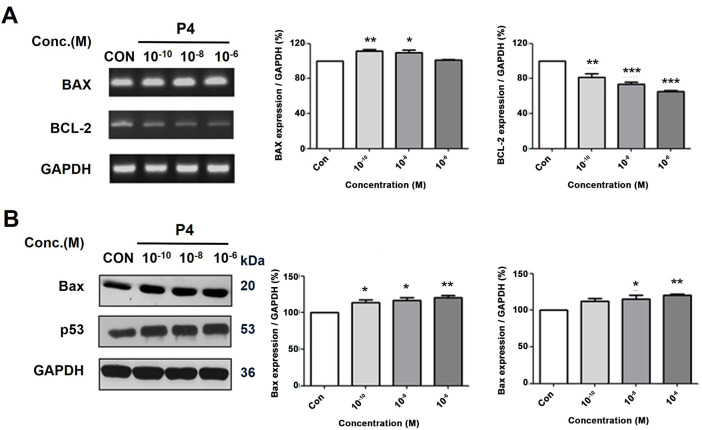
Altered mRNA and protein expressions of apoptosis-related and tumor suppressor genes following the treatment of P4 with E2. In all experiments, MCF-7 CV cells were treated with E2 (10^−8^ M) as a control in combination with P4 (10^−10^, 10^−8^, and 10^−6^ M). The mRNA and protein expression level was investigated by RT-PCR and western blot after 24 h and 48 h treatment. Data show (**A**) the alterations in mRNA expression of apoptosis-related genes (pro-apoptosis gene BAX and anti-apoptosis gene BCL-2) in MCF-7 CV cells by RT-PCR and (**B**) the alterations in protein expression of apoptosis-related and tumor suppressor genes (Bax and p53) in MCF-7 CV cells by western blot analysis. Data was quantified using Gel Doc 2000. Values are presented as the mean ± SD from at least three independent experiments. Asterisks denote a statistically significant difference compared to the control group (*p < 0.05, **p < 0.01 and ***p < 0.001).

Cell proliferation and cell death in multicellular organisms must be regulated for homeostasis. The interplay between cell cycle progression and programmed cell death involves the control of genes like p53, PUMA, NOXA, BAX, BCL-2, APAF-1, CASP 9, and CYCS [26]. The experimental data showed an increase in the expression of the pro-apoptosis gene *BAX* and a decrease in the *BCL-2* after treatment with P4, as seen in both mRNA and protein levels (Fig. 7A,B). Also, the protein levels observed revealed that the expression of the p53 (a tumor suppressor gene) was upregulated after the P4 treatment. The results indicate that P4 has the potential to induce apoptosis in MCF-7 CV breast cancer cells by modulating the BAX, p53, and BCL-2 genes. Consequently, this treatment potentially enhances apoptosis and hinders cancer cell survival in MCF-7 CV cells.

### 3.8 Functional Role of the PR in the EMT of MCF-7 CV Breast Cancer Cells

**Figure 8:**
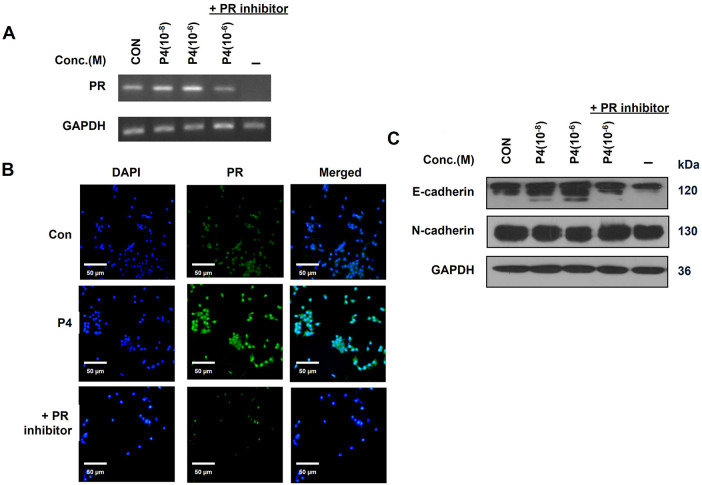
Role of PR in the inhibition of breast cancer migration. MCF-7 CV cells were treated with E2 (10^−8^ M) as a control in combination with P4 or/and RU486 at indicated concentrations in all experiments. (**A**) The level of mRNA expression of PR was investigated by RT-PCR after 24 h treatment. (**B**) The protein expression level of PR in MCF-7 CV cells was investigated by immunocytochemistry after 48 h treatment. (**C**) The alterations in protein expression of EMT-related genes (E-cadherin, N-cadherin) were investigated by western blot analysis after the treatment. Values are presented as the mean ± SD from at least three independent experiments. Asterisks denote a statistically significant difference compared to the control group.

MCF-7 CV cells were treated with a 10^−8^ M PR inhibitor (RU486) to investigate the role of PR in inhibition of breast cancer cells’ migration. At that concentration, RU486 did not affect cancer cell proliferation but blocked the effects of P4 without any side effects. The expression of the PR was increased by P4 (Fig. 8A,B), but in the presence of RU486 and P4, the PR expression was reduced to control levels. These results indicate that the P4 treatment induced the PR expression and that the co-treatment with RU486 (10^−8^ M) can sufficiently prevent the effects of P4. In addition, in the presence of the inhibitor, P4 did not affect EMT. In the presence of RU486, increases in the E-cadherin expression were reduced, and in contrast, N-cadherin expression, which was decreased by P4, was increased (Fig. 8C). Specifically, since the antibody recognizes the epitope near the N-terminal end of PR-B, it can only detect the isoform PR. Taken together, we identified PR as a critical mechanistic link in mediating EMT.

## 4 Discussion

The role of progesterone in breast cancer is complex and has been a subject of significant debate. While some clinical studies have linked progestogenic agents to an increased risk of breast cancer, there is also substantial evidence for a protective, anti-proliferative role, particularly for natural progesterone in an estrogen-rich environment. The clinical significance of the present study should be viewed in this context. Our work provides a mechanistic basis for the well-established clinical observation that PR expression is a strong favorable prognostic indicator in ER-positive tumors. The data presented here suggest that one reason for this better prognosis is that a functional PR pathway can directly counteract estrogen’s tumor-promoting effects by inhibiting the epithelial-mesenchymal transition, a key process in cancer cell invasion and metastasis.

The prevalence of ER-positive and PR-positive breast cancer cases has been reported to be 80% and 60%, respectively. Tumors that co-express ER and PR are more likely to exhibit favorable responses to therapies targeting hormone pathways than those lacking ER or PR expression [27]. Numerous indications suggest a strong correlation between elevated levels of estrogen and a heightened susceptibility to ER-positive breast cancer. This association is attributed to estrogen’s role in activating specific genes that promote cell growth and viability through the ER pathway [28]. Hence, the ER and its related signaling pathways have emerged as a significant focal point in the management of estrogen receptor-positive breast malignancies, resulting in a positive outlook, particularly for the ER^+^/PR^+^ breast cancer subtype, which generally shows better prognosis and improved responsiveness to endocrine therapies compared to other subtypes such as ER^+^/PR^−^, HER2^+^, and triple-negative (ER^−^/PR^−^/HER2^−^) breast cancers [29–31].

Nevertheless, the presence of progesterone in the context of breast cancer is a point of ongoing dispute within the scientific community. Despite the inhibitory impact of progesterone on the growth of breast and endometrial tumors, it is indicated by research that the use of progesterone in HRT might enhance the likelihood of developing breast cancer [32,33]. Furthermore, in clinical trials, progestin, a synthetic derivative of progesterone known for its similar physiological effects, has been employed in the treatment of advanced forms of breast cancer. On the contrary, there is a study indicating that exposure to progestin may increase the likelihood of breast cancer in both premenopausal and postmenopausal women [34,35]. These contradictory findings suggest the potential dual roles of progesterone, highlighting both protective and protumorigenic effects, and emphasize the necessity for cautious interpretation and context-dependent evaluation.

Nevertheless, the molecular mechanisms of progesterone for breast cancer inhibition have been well documented [36]. Progesterone has been reported to have a suppressive impact on the proliferation of human breast cancer cells by triggering cell cycle arrest via enhancing CDK inhibitors like p21, p27, and p18 [19,37]. In addition, progesterone exhibited a strong antiproliferative effect on T-47D breast cancer cells by inducing apoptosis through the upregulation of *p53* and downregulation of *Bcl-2* [18]. Likewise, in another study, the apoptosis induced by the PI3K/Akt pathway (inhibitor LY294002) was halted by progesterone [38]. In addition, there are some reports showing the attenuation of the E2 effect by P4 in hormone-responsive cancers. In uterine epithelial cells, the application of progesterone inhibited the cell proliferation induced by the E2 by suppressing CDK2, cyclin A, and cyclin E [39] or inhibiting the PI3K pathway and cyclin D1 expression [40].

Further exploration into the functions of progesterone in the advancement of breast cancer, this current research delved into the impacts of P4 on the EMT mechanism and the migration capacity of MCF-7 CV breast cancer cells. The process of EMT is essential for a wide range of biological activities such as embryonic growth, wound mending, tissue rejuvenation, and organ fibrosis [41]. It also plays a role in cancer progression. In cancer, the epithelial cells undergo a dramatic remodeling of the cytoskeletal components through the EMT process and finally acquire mesenchymal characteristics and manifest a migratory phenotype, which permits cancer cells acquiring mesenchymal features to escape from the primary tumor to secondary sites [42,43]. Since the acquisition of migratory and invasive phenotypes by cancer cells is considered the first step in metastatic dissemination, EMT eventually contributes to tumor progression and metastasis [44].

Protein markers expressed by different cell types can help understand how cancer cells undergo epithelial-mesenchymal transition (EMT) [45]. For example, epithelial cells have a lot of E-cadherin, a transmembrane protein essential for the adherens junction, and mesenchymal cells have N-cadherin, fibronectin, and vimentin, markers required for cellular migration. In addition, many transcription factors are thought to play a significant role in initiating EMT, including Snail1, Snail2 (also known as Slug), ZEB1, and ZEB2 [46].

Concerning the increase in the moving ability of mesenchymal cells formed from epithelial cells through the EMT process, proteolytic enzymes, MMPs, and cathepsins play a crucial role in the enhancement of the invasiveness of cancer cells [47]. MMPs are endopeptidases that contain zinc and rely on calcium for their function. They play a crucial role in breaking down the extracellular matrix to aid in tumor cell migration [48]. Among the several types of MMPs, MMP-2 and -9 are mostly regarded as necessary in migration [49]. Cathepsins are enzymes responsible for protein breakdown and are typically activated in the acidic environment of lysosomes, playing a vital role in the turnover of mammalian cells [50]. Cathepsins B and L are associated with matrix degradation and the invasion of cancer cells [51].

The current study examined if P4 inhibits E2-induced cell activities in breast cancer cells. E2 was found to promote breast cancer development by activating cell proliferation, EMT, and migration. P4 inhibits cell proliferation and down-regulate cyclin D1 and E1 in these MCF-7 CV cells. These proteins activate cyclin-dependent kinase (CDK) enzymes essential for cell cycle progression. In agreement with prior research, P4 induces apoptosis in MCF-7 CV cells by reducing Bcl-2 levels and increasing Bax and p53 levels. P4 suppresses key markers associated with EMT in an estrogenic environment by affecting the expression of vimentin, snail, slug, and E-cadherin. P4 inhibits EMT in MCF-7 CV cells by decreasing MMP-9 and cathepsin B in the presence of E2. Nevertheless, upon co-administration with E2, RU486 reinstated the cellular migration and invasion that had previously been suppressed by P4 to a state resembling control levels. Moreover, it also reversed the decreased activity of proteases induced by P4 back to control levels. These findings suggest the involvement of PR in the P4-triggered impediment of the migration and invasion of MCF-7 CV cells.

Collectively, the results of this study suggest that P4 can decrease the migration potential induced by E2 in human epithelial breast cancer cells. PR-mediated suppression of EMT might be particularly relevant in ER^+^/PR^+^ breast cancer subtypes, which typically exhibit more favorable clinical outcomes [52,53].

**Figure 9:**
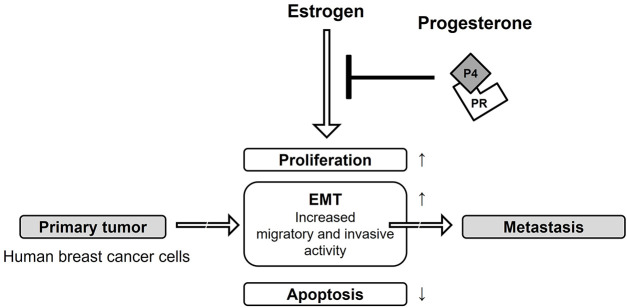
Protection effect of progesterone on E2-induced MCF-7 CV breast cancer progression. The aim of the current study was to assess the additional influence of P4 on cell proliferation, apoptosis, EMT and migratory and invasive characteristics of E2-induced MCF-7 CV breast cancer cells that express both estrogen and progesterone receptors. It was found that upon treatment with P4, there was reduced cell proliferation of MCF-7 CV breast cancer cells treated with E2 due to decreased expression of Cyclin D1 and E1 proteins. In addition, treatment with P4 stimulated apoptosis among E2-treated MCF-7 CV breast cancer cells due to increased expression of Bax and p53 proteins, but a decrease in expression of Bcl-2 protein. Furthermore, the data suggests that E2 treatment previously stimulated an EMT-like invasive behavior in MCF-7 CV breast cancer cells; however, treatment with P4 reversed this behavior by increasing E-cadherin mRNA and protein expression, and decreasing N-cadherin, vimentin and transcription factors (e.g., snail, slug) associated with an EMT. Similarly, P4 treatment inhibited the E2-stimulated migration and invasion of MCF-7 CV breast cancer cells through the reduction of proteolytic enzyme (e.g., MMP-9 and cathepsin B) protein expression. When MCF-7 CV cells were treated with both P4 and RU486 (a specific PR inhibitor), the reduction in migration and invasion levels associated with P4 treatment were restored to control levels. Thus, the observed effects of P4 in inhibiting migration and invasion of MCF-7 CV breast cancer cells, which were found to be upregulated by E2, are mediated by PR. The present study did not investigate the direct interactions between the P4 and E2 receptor pathways; however, it has been previously shown that P4 exposures regulate ER expression and transcriptional activity and thus represent another major mechanism by which P4 displays its anti-estrogenic properties. Therefore, examining whether the inhibitory actions of P4 observed in our study are also mediated by a downregulation of ER levels or function is a significant limitation and a critical direction for future research to fully map the interplay between these hormone signaling axes.

This study provides important insights into how progesterone signaling, mediated through PR, can inhibit processes related to breast cancer progression. While our results establish the integral role of PR in opposing estrogen-driven effects, the specific contributions of its isoforms, PR-A and PR-B, were not determined in this study. Understanding the distinct functions of each isoform in regulating EMT is a critical area for future research. Subsequent studies will be needed to dissect their individual roles, which could ultimately inform the development of more targeted therapies for ER+/PR+ breast cancer. These effects include suppression of proliferation, EMT, migration, and invasion, as well as induction of apoptosis, as summarized in Fig. 9. Most of all, the present study was the first to identify that P4 treatment can suppress E2-induced cancer progression by causing an obvious inhibition of the EMT along with the migration and invasion of breast cancer cells. Although the mechanisms associated with the attenuation of the E2-mediating gene expression by P4 in breast cancer are complex and still incompletely characterized, progesterone plays a crucial role in breast cancer through PRs that act as transcriptional factors to regulate several target genes directing cell proliferation, growth, motility, and invasion of cancer cells. Our findings show that progesterone inhibits estrogen-induced EMT and migration in ER^+^/PR^+^ breast cancer cells via EMT-related markers. However, this study did not directly evaluate downstream pathways such as PI3K/Akt or MAPK. However, these pathways are known to interact with ER/PR signaling and play critical roles in regulating proliferation, survival, and EMT in hormone-responsive cancers. Highlighting this limitation, we have added a statement to emphasize that investigating these signaling cascades represents a crucial direction for future studies to expand the mechanistic understanding of progesterone’s actions in breast cancer.

We posit that P4 treatment may serve as a potential clinical strategy to prevent breast cancer progression. A key limitation of our study design should also be noted. Our experiments consistently used estrogen-treated cells as the baseline control against which the effects of progesterone were measured. This approach was chosen to directly test our primary hypothesis regarding the antagonistic action of P4 in an estrogenic environment. However, this design does not include a true untreated control (lacking both E2 and P4). Consequently, while our results robustly demonstrate that P4 can inhibit proliferation and migration relative to an E2-treated state, they do not allow for the quantification of the full inductive effect of E2 compared to a basal, hormone-deprived state. Future studies incorporating such controls would be valuable for a more comprehensive understanding.

Furthermore, the observations derived from a single cell line should be cautiously interpreted. We acknowledge that different ER+/PR+ cell lines can exhibit distinct hormonal sensitivities; for example, T47D cells have been reported to be highly responsive to progesterone, in some cases more so than MCF-7 cells. Therefore, validating our findings in other relevant models, such as the T47D cell line, is a critical next step to determine the broader applicability of our conclusions and will be a focus of our future work. Furthermore, validating these *in vitro* findings by examining these marker expressions in clinical ER+/PR+ breast cancer tissue specimens is a crucial future step to confirm their relevance to human disease. It is also important to consider the concentrations of hormones used in our *in vitro* model. The selected concentrations (e.g., 10 nM E2) are consistent with those widely used in the literature to study hormonal responses in breast cancer cell lines [54,55]. While these concentrations may be higher than average circulating physiological levels, they are often necessary to elicit a robust and reproducible biological response in a cell culture system that lacks the complex metabolic and systemic factors present *in vivo*. Further consideration of the therapeutic and preventive strategies using P4 against breast cancer is still needed due to the controversial issues associated with HRT, including P4.

## Data Availability

The data that support the findings of this study are available from the Corresponding Author, Kyung-Chul Choi, upon reasonable request.
